# Supplementary material for: Conformational trajectory of allosteric gating of the human cone photoreceptor cyclic nucleotide-gated channel
Source: Nat Commun. 2023 Jul 18;14:4284. doi: 10.1038/s41467-023-39971-8 (PMC10354024; doi:10.1038/s41467-023-39971-8)
Supplement: Supplementary file 1 — Supplementary Information [file 41467_2023_39971_MOESM1_ESM.pdf]

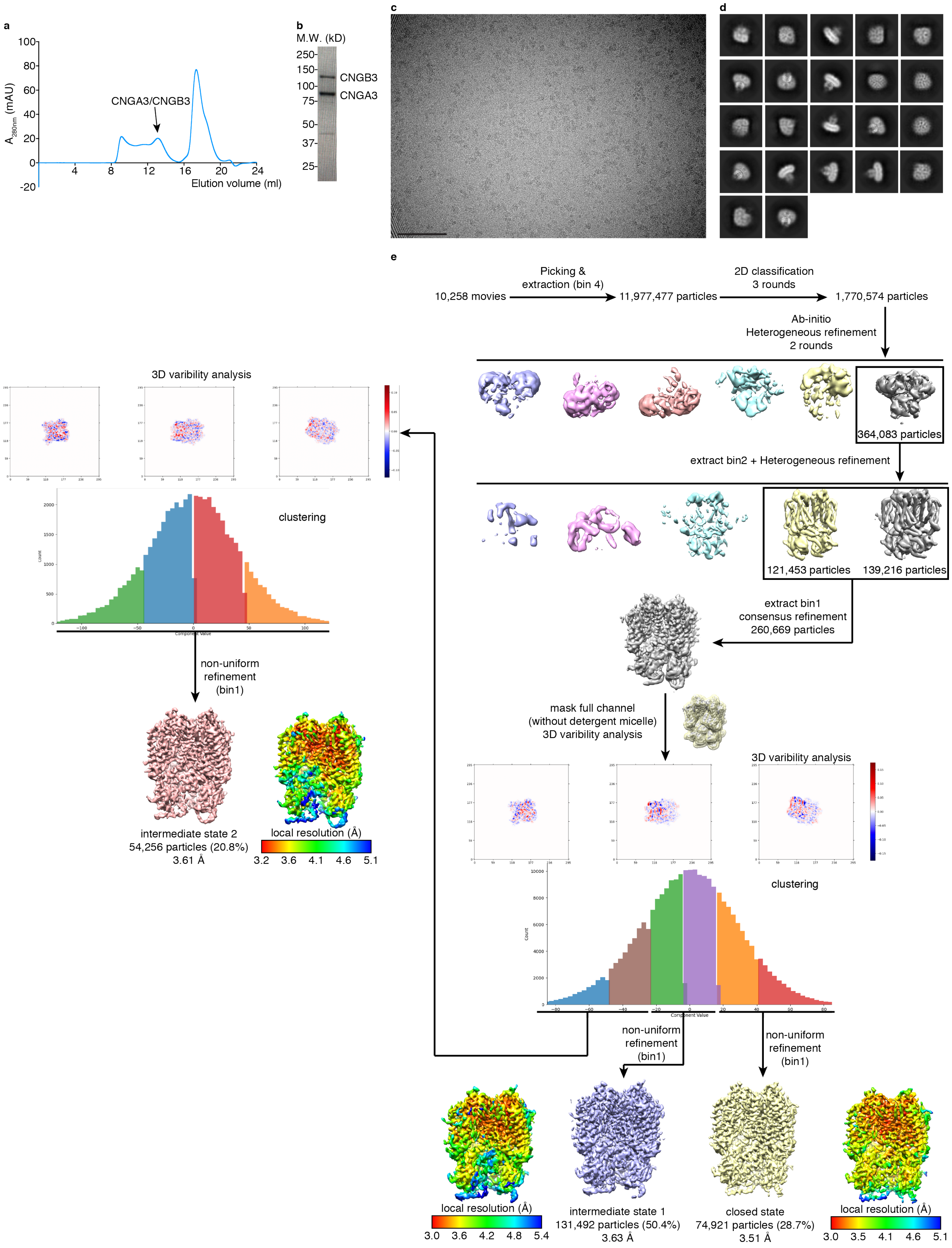
a
100
80
60
40
20
0
-20
A_{280nm} (mAU)
CNGA3/CNGB3
4
8
12
16
20
24
Elution volume (ml)
b
M.W. (kD)
250
150
100
75
50
37
25
CNGB3
CNGA3
c
d
e
10,258 movies
Picking & extraction (bin 4)
11,977,477 particles
2D classification 3 rounds
1,770,574 particles
Ab-initio Heterogeneous refinement 2 rounds
364,083 particles
extract bin2 + Heterogeneous refinement
121,453 particles
139,216 particles
extract bin1 consensus refinement 260,669 particles
mask full channel (without detergent micelle) 3D varibility analysis
3D varibility analysis
clustering
non-uniform refinement (bin1)
non-uniform refinement (bin1)
local resolution (Å)
3.0
3.6
4.2
4.8
5.4
intermediate state 1
131,492 particles (50.4%)
3.63 Å
closed state
74,921 particles (28.7%)
3.51 Å
local resolution (Å)
3.0
3.5
4.1
4.6
5.1
3D varibility analysis
clustering
non-uniform refinement (bin1)
intermediate state 2
54,256 particles (20.8%)
3.61 Å
local resolution (Å)
3.2
3.6
4.1
4.6
5.1

Supplementary Fig. 1 | Cryo-EM single-particle analysis of cGMP-bound full-length CNGA3/CNGB3 in GDN, Part I. a, Gel filtration curve of full-length CNGA3/CNGB3 in GDN. **b**, SDS-PAGE of full-length CNGA3/CNGB3 used for cryo-EM. Uncropped gel image is available in Source Data file. Protein purification was performed at least twice and SDS-PAGE results were similar. **c**, Representative motion-corrected micrograph. Scale bar: 100 µm. Cryo-EM data collection was performed once. **d**, Gallery of typical averages from 2D classification. **e**, Flow chart of cryo-EM image processing. See Methods for details.

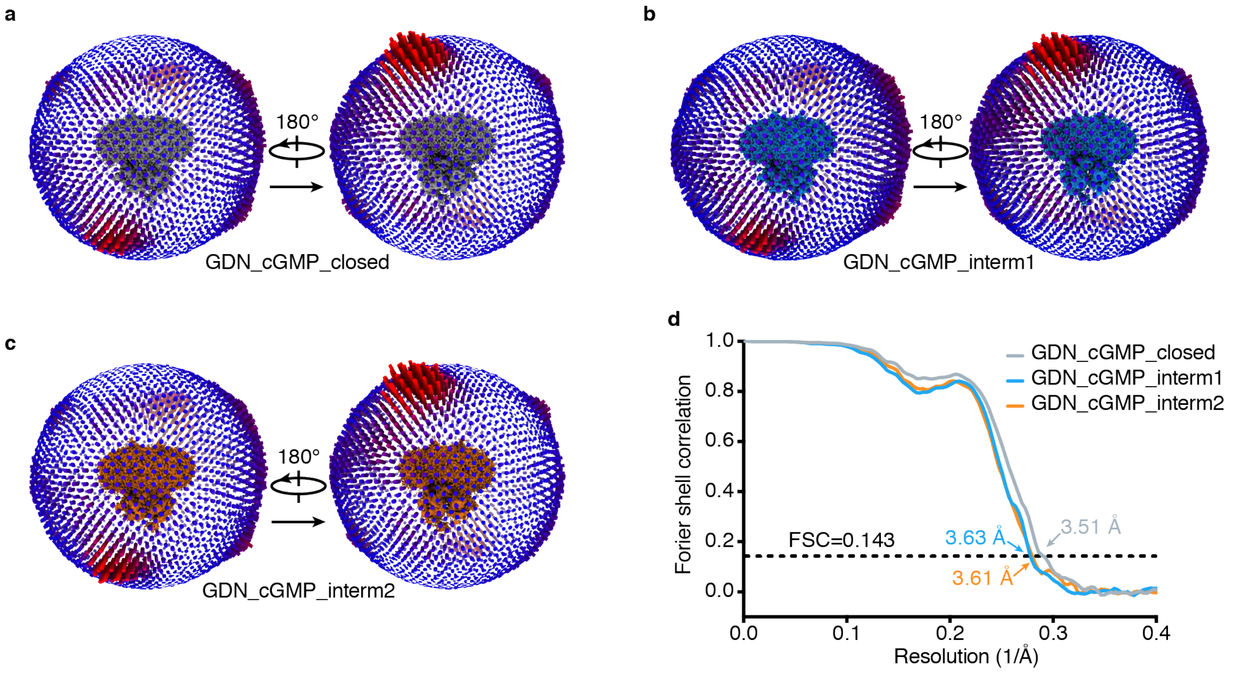

Supplementary Fig. 2 | Cryo-EM single-particle analysis of cGMP-bound full-length CNGA3/CNGB3 in GDN, Part II. a-c, Euler angle distributions of particles used in the final 3D reconstruction with C1 symmetry of GDN_cGMP_closed state (**a**), GDN_cGMP_intermediate1 state (**b**), and GDN_cGMP_intermediate2 state (**c**). **d**, Gold-standard FSC curves of the final 3D reconstructions of the three states.

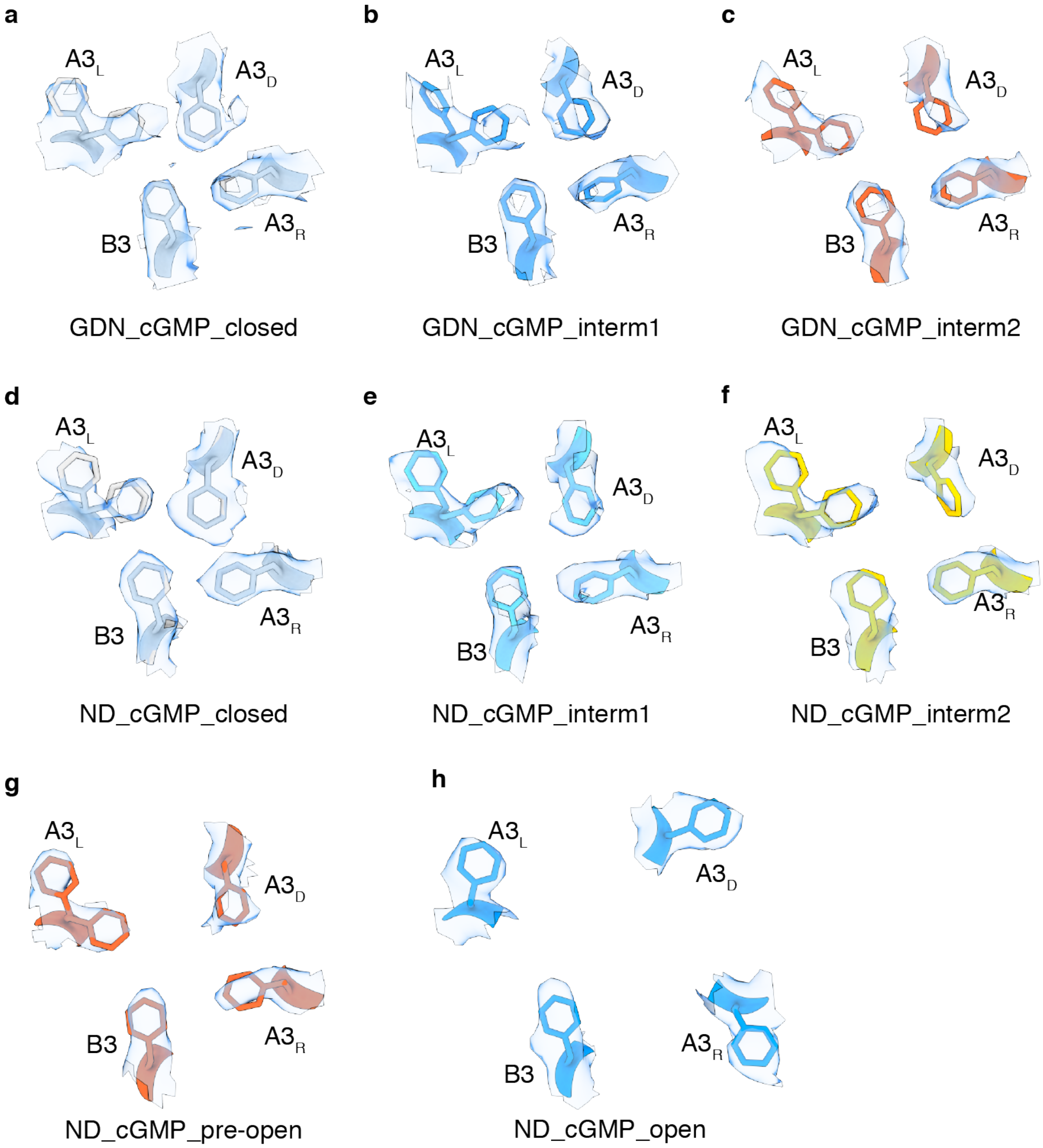

Supplementary Fig. 3 | Cryo-EM density maps of F392 of CNGA3 and F434 of CNGB3 in the indicated structures. Local cryo-EM density maps, all contoured to 6σ, are shown as transparent blobs and superimposed with the modeled amino acid. **a-c,** In GDN. **d-h,** In lipid nanodisc.

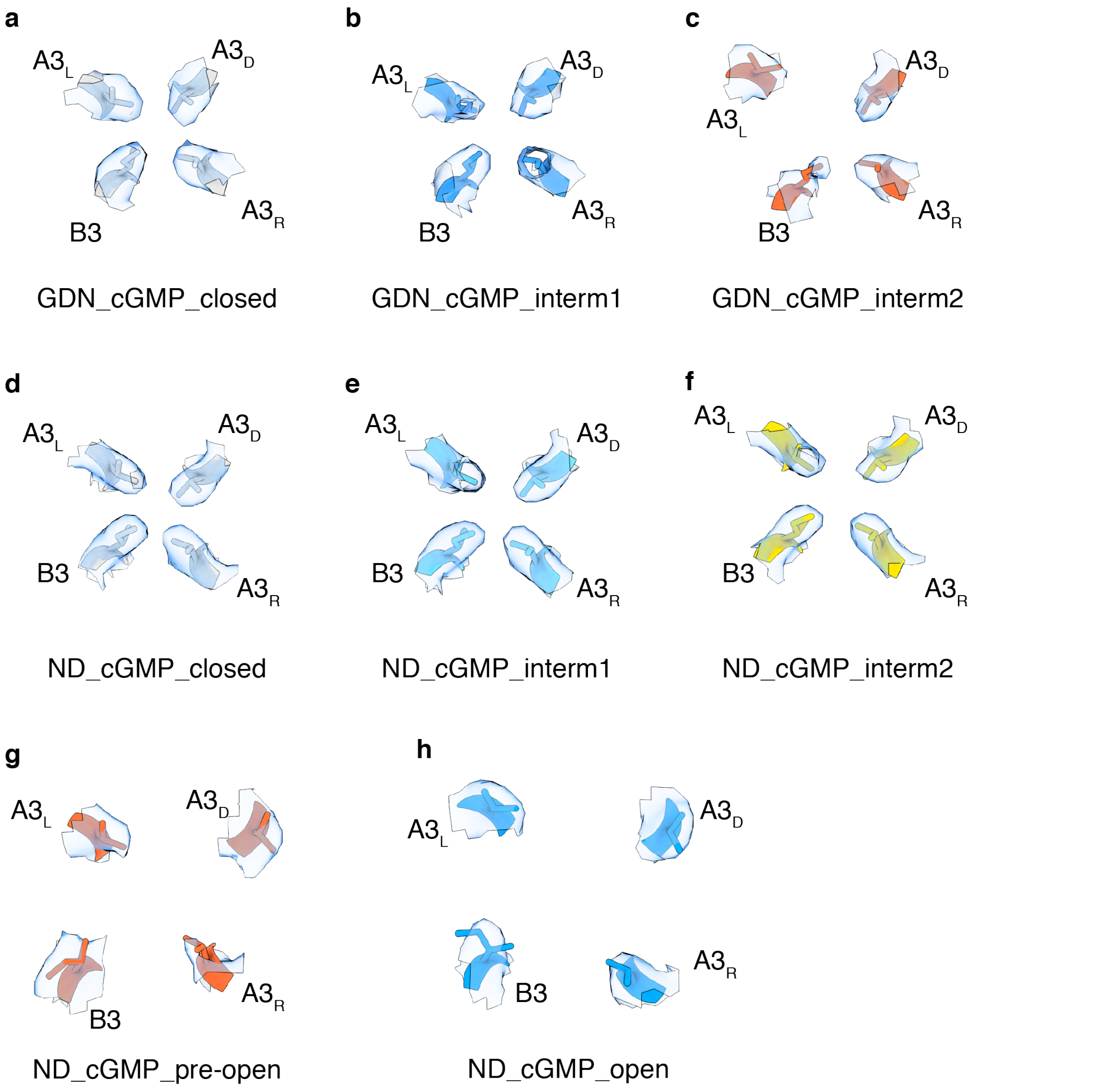

Supplementary Fig. 4 | Cryo-EM density maps of V396 of CNGA3 and I438 of CNGB3 in the indicated structures. Local cryo-EM density maps, all contoured to 6σ, are shown as transparent blobs and superimposed with the modeled amino acid. **a-c,** In GDN. **d-h,** In lipid nanodisc.

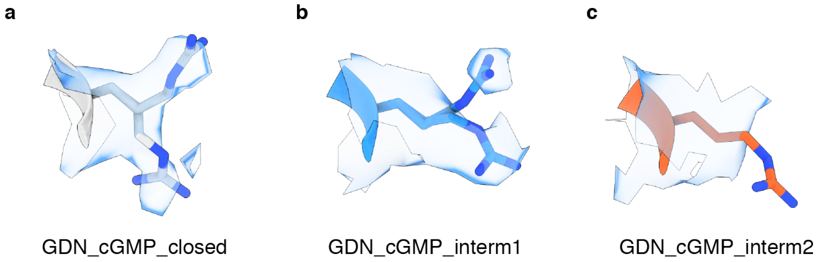

Supplementary Fig. 5 | Cryo-EM density maps of R442 of CNGB3 in the indicated structures. Local cryo-EM density maps, all contoured to 6σ, are shown as transparent blobs and superimposed with the modeled amino acid.

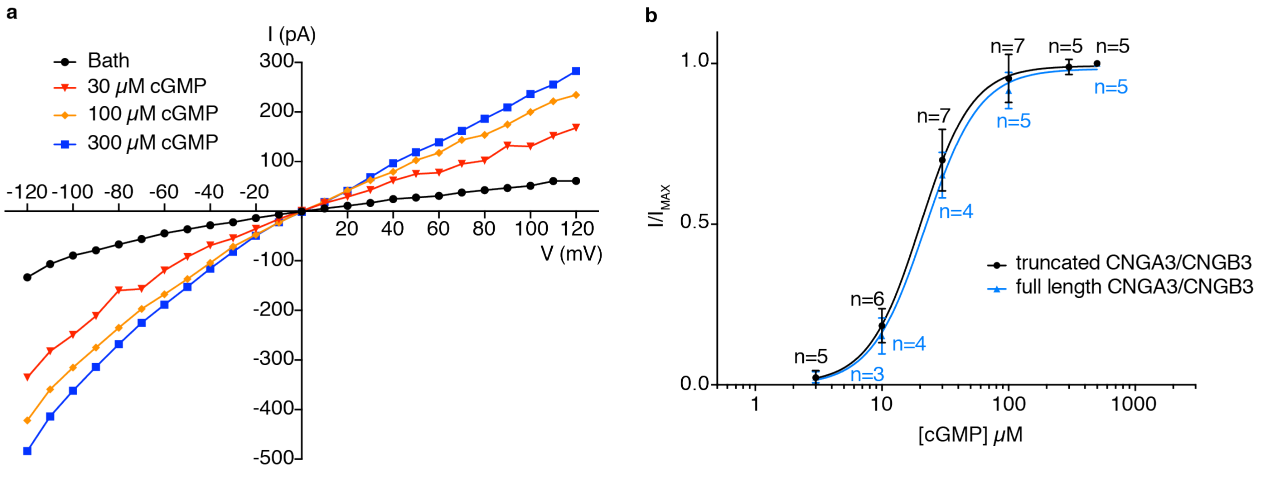

Supplementary Fig. 6 | Functional analysis of N-terminally truncated CNGA3/CNGB3. a, Representative current-voltage (I-V) curves of truncated CNGA3/CNGB3. Macroscopic currents were recorded in the same inside-out patch obtained from a HEK 293T cell expressing the trunCNGA3-P2A-trunCNGB3 construct in the absence or presence of the indicated concentrations of cGMP. Currents at -100 mV were used to generate the cGMP dose-response curve in (**b**). **b,** Dose-response curves of cGMP activation of full-length and truncated CNGA3/CNGB3 at -100 mV. Data points represent mean ± SD of the indicated number of independent measurements. Curves represent fit to the Hill equation in the form of $I(X)=X^n/(X^n+EC_{50}^n)$, where $I(X)$ is the normalized current, X the cGMP concentration, n the Hill coefficient, and EC_{50} the cGMP concentration producing half maximal current. The fit yields an EC_{50} of 20 μM and 22 μM, respectively, for the full-length and truncated CNGA3/CNGB3, with n of 2.1 and 2.0. Source Data file is provided.

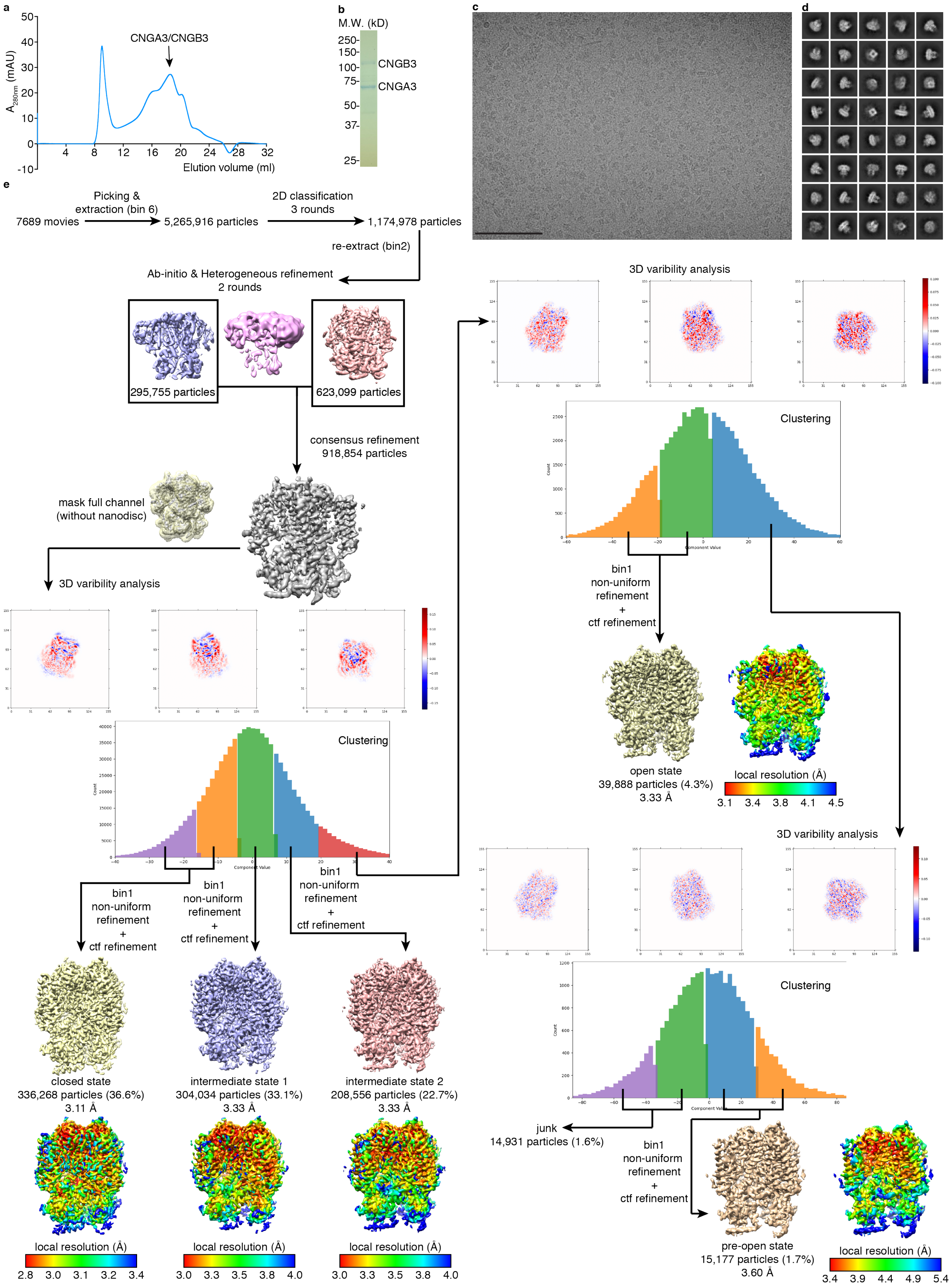
a
CNGA3/CNGB3
A280nm (mAU)
Elution volume (ml)
b
M.W. (kD)
CNGB3
CNGA3
c
d
e
Picking &
extraction (bin 6)
7689 movies
5,265,916 particles
2D classification
3 rounds
1,174,978 particles
re-extract (bin2)
Ab-initio & Heterogeneous refinement
2 rounds
295,755 particles
623,099 particles
consensus refinement
918,854 particles
mask full channel
(without nanodisc)
3D varibility analysis
Clustering
bin1
non-uniform
refinement
+
ctf refinement
closed state
336,268 particles (36.6%)
3.11 Å
intermediate state 1
304,034 particles (33.1%)
3.33 Å
intermediate state 2
208,556 particles (22.7%)
3.33 Å
local resolution (Å)
2.8 3.0 3.1 3.2 3.4
3.0 3.3 3.5 3.8 4.0
open state
39,888 particles (4.3%)
3.33 Å
3.1 3.4 3.8 4.1 4.5
junk
14,931 particles (1.6%)
pre-open state
15,177 particles (1.7%)
3.60 Å
3.4 3.9 4.4 4.9 5.4

Supplementary Fig. 7 | Cryo-EM single-particle analysis of cGMP-bound N-terminally truncated CNGA3/CNGB3 in POPG/POPC nanodiscs, Part I. a, Gel filtration curve of truncated CNGA3/CNGB3 in 1:1 POPG:POPC nanodisc. **b**, SDS-PAGE of truncated CNGA3/CNGB3 used for cryo-EM. Uncropped gel image is available in Source Data file. Protein purification was performed at least twice and SDS-PAGE results are similar. **c**, Representative motion-corrected micrograph. Scale bar: 100 μm. Cryo-EM data collection was performed once. **d**, Gallery of typical averages from 2D classification. **e**, Flow chart of cryo-EM image processing. See Methods for details.

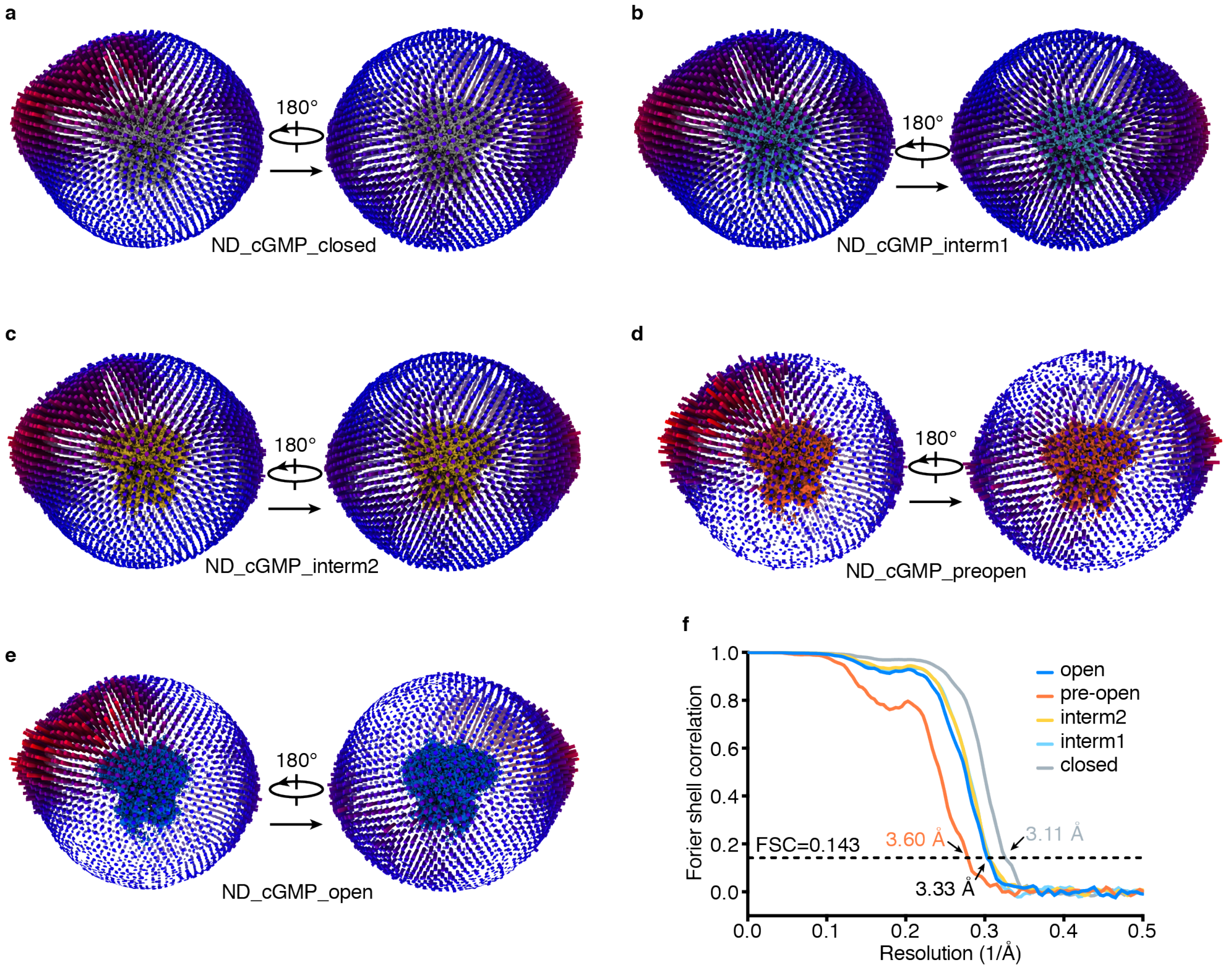

Supplementary Fig. 8 | Cryo-EM single-particle analysis of cGMP-bound N-terminally truncated CNGA3/CNGB3 in POPG/POPC nanodiscs, Part II. a-e, Euler angle distributions of particles used in the final 3D reconstruction with C1 symmetry of ND_cGMP_closed state (**a**), ND_cGMP_intermediate1 state (**b**), ND_cGMP_intermediate2 state (**c**), ND_cGMP_pre-open state (**d**), and ND_cGMP_open state (**e**). **f**, Gold-standard FSC curves of the final 3D reconstructions of the five states.

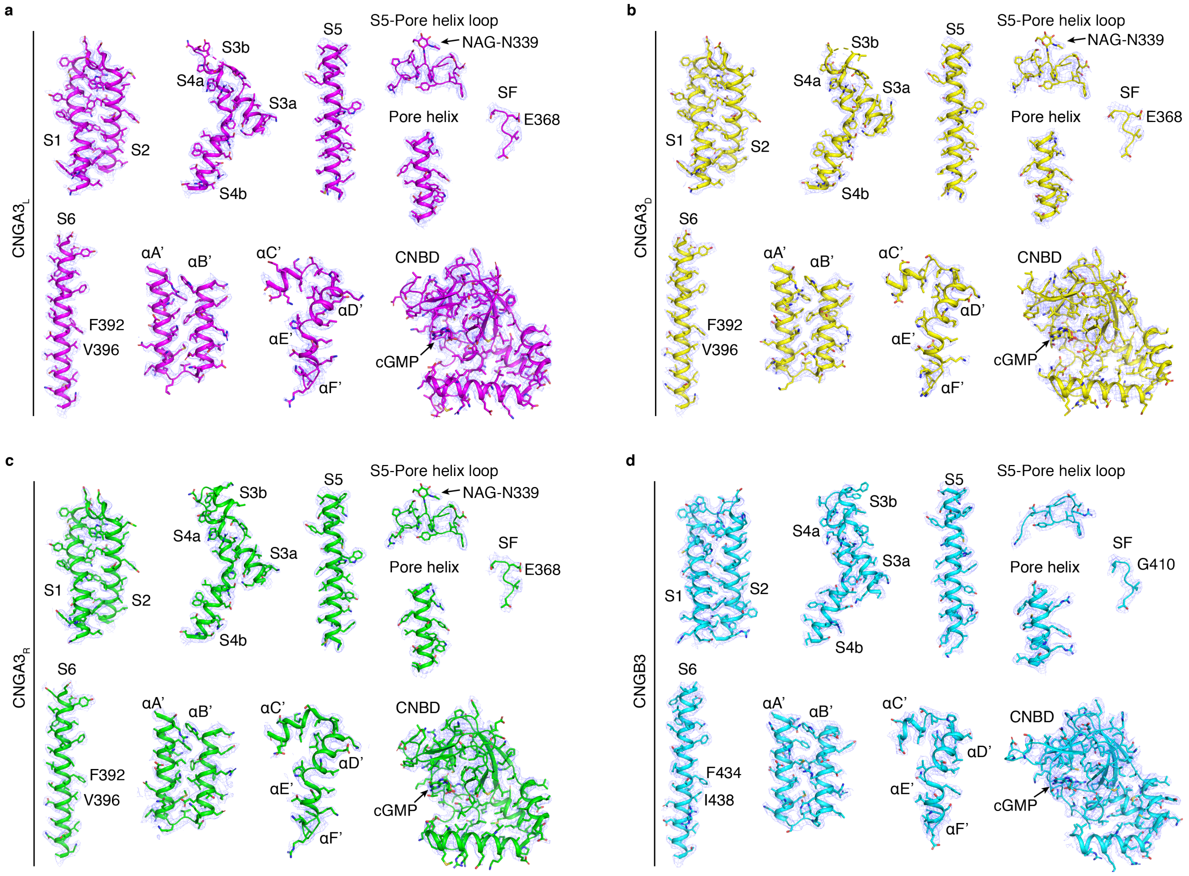

Supplementary Fig. 9 | Cryo-EM density maps and fitting of atomic models of selected key regions in all four subunits of cGMP-bound closed-state CNGA3/CNGB3 in POPG/POPC nanodiscs. All maps were low-pass filtered to 3.11 Å, sharpened with a temperature factor of −117.1 Å^2 and contoured at 3σ. NAG: N-acetyl-beta-D-glucosamine.

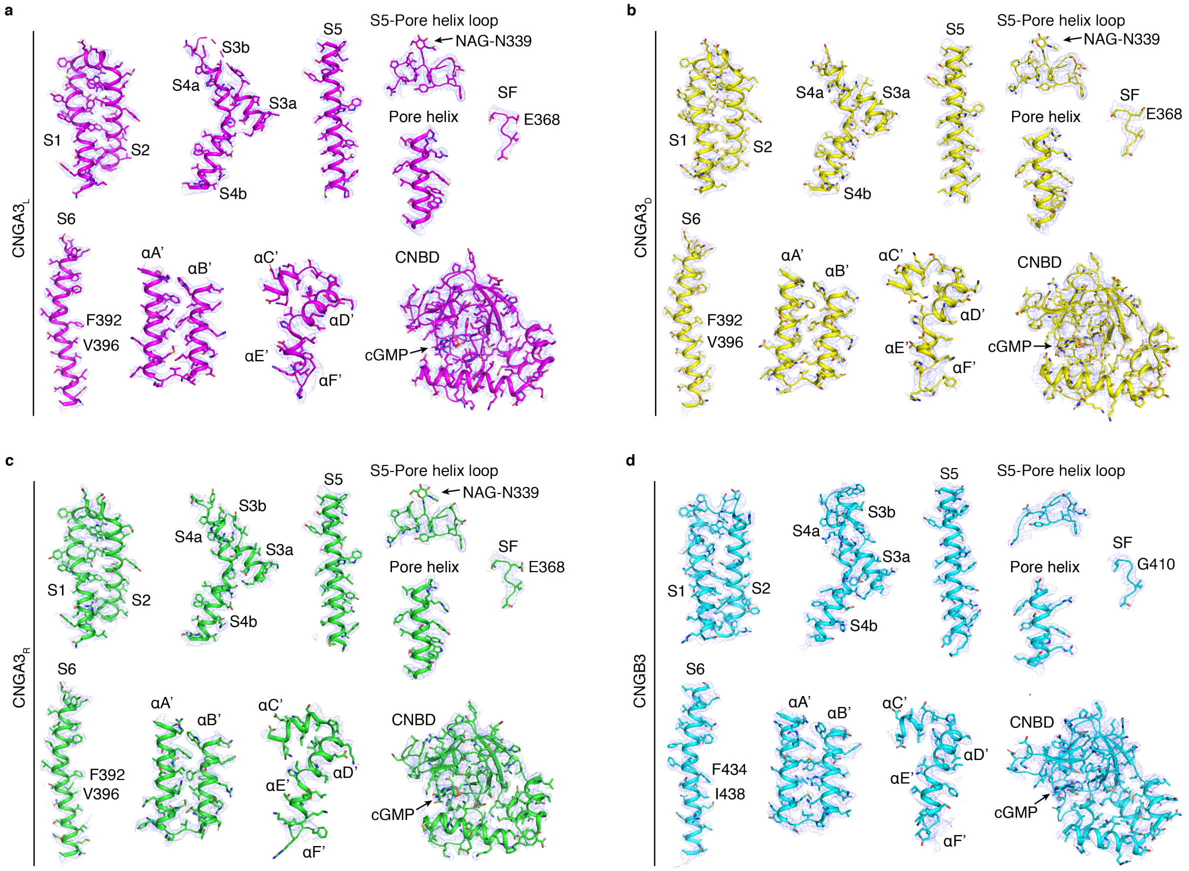

Supplementary Fig. 10 | Cryo-EM density maps and fitting of atomic models of selected key regions in all four subunits of cGMP-bound open-state CNGA3/CNGB3 in POPG/POPC nanodiscs. All maps were low-pass filtered to 3.33 Å, sharpened with a temperature factor of −78.8 Å^2 and contoured at 3σ. NAG: N-acetyl-beta-D-glucosamine.
