# Supplementary material for: Conformational trajectory of allosteric gating of the human cone photoreceptor cyclic nucleotide-gated channel
Source: Nat Commun. 2023 Jul 18;14:4284. doi: 10.1038/s41467-023-39971-8 (PMC10354024; doi:10.1038/s41467-023-39971-8)
Supplement: Supplementary file 3 — Description of Additional Supplementary Files [file 41467_2023_39971_MOESM3_ESM.pdf]

Description of Additional Supplementary Files

File name: Supplementary Movie 1
Description: Conformational changes of cGMP-bound CNGA3/CNGB3 in GDN. The movie is generated from 3D variability analysis and shows a continuum of 13 different 3D density maps generated from 99% of the particles used to produce the three states in GDN. Each subunit is color coded as in Fig. 2a. The channel is first viewed parallel to the membrane (side view) and then from the intracellular side (bottom-up view).

File name: Supplementary Movie 2
Description: Conformational changes of cGMP-bound CNGA3/CNGB3 in POPG/POPC nanodiscs. The movie is generated from 3D variability analysis and shows a continuum of 13 different 3D density maps generated from 99.4% of the particles used to produce the five states in POPG/POPC nanodiscs. Each subunit is color coded as in Fig. 2a. The channel is first viewed parallel to the membrane (side view) and then from the intracellular side (bottom-up view). Notice that each subunit undergoes different movements.
