# Supplementary material for: Conformational trajectory of allosteric gating of the human cone photoreceptor cyclic nucleotide-gated channel
Source: Nat Commun. 2023 Jul 18;14:4284. doi: 10.1038/s41467-023-39971-8 (PMC10354024; doi:10.1038/s41467-023-39971-8)
Supplement: Supplementary file 6 — Reporting Summary [file 41467_2023_39971_MOESM6_ESM.pdf]

nature portfolio

Corresponding author(s): Jian Yang
Last updated by author(s): Jun 11, 2023

Reporting Summary

Nature Portfolio wishes to improve the reproducibility of the work that we publish. This form provides structure for consistency and transparency in reporting. For further information on Nature Portfolio policies, see our Editorial Policies and the Editorial Policy Checklist.

Statistics

For all statistical analyses, confirm that the following items are present in the figure legend, table legend, main text, or Methods section.

n/a	Confirmed	
☐	☒	The exact sample size (n) for each experimental group/condition, given as a discrete number and unit of measurement
☐	☒	A statement on whether measurements were taken from distinct samples or whether the same sample was measured repeatedly
☒	☐	The statistical test(s) used AND whether they are one- or two-sided<br>*Only common tests should be described solely by name; describe more complex techniques in the Methods section.*
☒	☐	A description of all covariates tested
☒	☐	A description of any assumptions or corrections, such as tests of normality and adjustment for multiple comparisons
☐	☒	A full description of the statistical parameters including central tendency (e.g. means) or other basic estimates (e.g. regression coefficient) AND variation (e.g. standard deviation) or associated estimates of uncertainty (e.g. confidence intervals)
☒	☐	For null hypothesis testing, the test statistic (e.g. F, t, r) with confidence intervals, effect sizes, degrees of freedom and P value noted<br>*Give P values as exact values whenever suitable.*
☒	☐	For Bayesian analysis, information on the choice of priors and Markov chain Monte Carlo settings
☒	☐	For hierarchical and complex designs, identification of the appropriate level for tests and full reporting of outcomes
☒	☐	Estimates of effect sizes (e.g. Cohen's d, Pearson's r), indicating how they were calculated

Our web collection on statistics for biologists contains articles on many of the points above.

Software and code

Policy information about availability of computer code

Data collection	LEGINON 3.5, pCLAMP 8.2
Data analysis	MotionCor2, GCTF v1.06, RELION 3.0.7, cryoSPARC 3.3.2, Microsoft Excel v15.26, UCSF Chimera 1.16, UCSF ChimeraX 1.15, COOT 0.9 EL, Phenix-1.15.2-3472, PyMol 2.3.2, MolProbity 4.5, HOLE 2.2.005, Prism 7, iMovie 10.1.1

For manuscripts utilizing custom algorithms or software that are central to the research but not yet described in published literature, software must be made available to editors and reviewers. We strongly encourage code deposition in a community repository (e.g. GitHub). See the Nature Portfolio guidelines for submitting code & software for further information.

Data

Policy information about availability of data

All manuscripts must include a data availability statement. This statement should provide the following information, where applicable:

- Accession codes, unique identifiers, or web links for publicly available datasets
- A description of any restrictions on data availability
- For clinical datasets or third party data, please ensure that the statement adheres to our policy

The authors declare that the data supporting the findings of this study are available within the paper. Full-length sequences of CNGA3 and CNGB3 are available at National Center for Biotechnology Information (NCBI) with reference code NP_001289.1 [https://www.ncbi.nlm.nih.gov/protein/NP_001289.1] and NP_061971.3 [https://www.ncbi.nlm.nih.gov/protein/NP_001289.1], respectively. The cryo-EM density maps generated in this study have been deposited in the Electron Microscopy Data Bank (https://www.ebi.ac.uk/pdbe/emdb/). For cGMP-bound full-length CNGA3/CNGB3 in GDN, the accession numbers for closed state,

intermediate state 1, and intermediate state 2 are EMD-28595 [https://www.ebi.ac.uk/emdb/EMD-28595], EMD-28603 [https://www.ebi.ac.uk/emdb/EMD-28603], and EMD-28611 [https://www.ebi.ac.uk/emdb/EMD-28611], respectively. For cGMP-bound truncated CNGA3/CNGB3 in POPG/POPC nanodiscs, the accession numbers for closed state, intermediate state 1, intermediate state 2, pre-open state, and open state are EMD-28622 [https://www.ebi.ac.uk/emdb/EMD-28622], EMD-28623 [https://www.ebi.ac.uk/emdb/EMD-28623], EMD-28624 [https://www.ebi.ac.uk/emdb/EMD-28624], EMD-28625 [https://www.ebi.ac.uk/emdb/EMD-28625], and EMD-28626 [https://www.ebi.ac.uk/emdb/EMD-28626], respectively. The coordinates of the atomic models generated by this study have been deposited in the Protein Data Bank (http://www.rcsb.org). For cGMP-bound full-length CNGA3/CNGB3 in GDN, the accession numbers for closed state, intermediate state 1, and intermediate state 2 are 8ETP [https://doi.org/10.2210/pdb8ETP/pdb], 8EU3 [https://doi.org/10.2210/pdb8EU3/pdb], and 8EUC [https://doi.org/10.2210/pdb8EUC/pdb], respectively. For cGMP-bound truncated CNGA3/CNGB3 in POPG/POPC nanodiscs, the accession numbers for closed state, intermediate state 1, intermediate state 2, pre-open state, and open state are 8EV8 [https://doi.org/10.2210/pdb8EV8/pdb], 8EV9 [https://doi.org/10.2210/pdb8EV9/pdb], 8EVA [https://doi.org/10.2210/pdb8EVA/pdb], 8EVB [https://doi.org/10.2210/pdb8evb/pdb], and 8EVC [https://doi.org/10.2210/pdb8evc/pdb], respectively. The atomic model for apo closed state CNGA3/CNGB3 in GDN (PDB: 7RHS [https://doi.org/10.2210/pdb7RHS/pdb]) and corresponding cryo-EM map (EMDB: 24468 [https://www.ebi.ac.uk/emdb/EMD-24468]) are used for comparison and analysis. The mass spec data, electrophysiological data, and unprocessed SDS-PAGE images are available as Source Data file online.

Human research participants

Policy information about studies involving human research participants and Sex and Gender in Research.

Reporting on sex and gender	Sex and gender are not relevant to this study.
Population characteristics	There are no human research participants in this study.
Recruitment	There are no human research participants in this study.
Ethics oversight	There are no human research participants in this study.

Note that full information on the approval of the study protocol must also be provided in the manuscript.

Field-specific reporting

Please select the one below that is the best fit for your research. If you are not sure, read the appropriate sections before making your selection.

☒ Life sciences ☐ Behavioural & social sciences ☐ Ecological, evolutionary & environmental sciences

For a reference copy of the document with all sections, see nature.com/documents/nr-reporting-summary-flat.pdf

Life sciences study design

All studies must disclose on these points even when the disclosure is negative.

Sample size	Sample sizes were not pre-determined. Cryo-EM images were collected until structures of satisfactory quality were solved, which suggested sufficient sample size. For inside-out patch recordings to generate cGMP dose response curves, multiple patches were recorded until each data point on the curve has at least 3 repeats and a curve fit with reasonable errors can be achieved. For full-length CNGA3/CNGB3, 5 patches were enough to achieve satisfactory result. For truncated CNGA3/CNGB3, 9 patches were enough.
Data exclusions	No data were excluded during structural analysis. For inside-out recordings, 4 patches were excluded because the basal current was too large.
Replication	Protein purification of both nanodisc and GDN sample was performed at least twice. All repeats have similar SDS-PAGE results and gel filtration curves. Cryo-EM data collection was only performed once for both GDN and nanodisc samples. Single particle analysis was performed 2 to 3 times independently using RELION or cryoSPARC. Similar results were observed. For full-length CNGA3/CNGB3 inside-out patch recordings, a total of 10 patches were recorded. Out of the 10, 6 have similar currents, and 1 patch has too large basal currents and was discarded. For truncated CNGA3/CNGB3, a total of 18 patches were recorded. Out of the 18, 12 have similar currents, and 3 patches has too large basal currents and was discarded. Mass spec experiment was performed only once.
Randomization	No group allocation was performed in structural experiments and electrophysiology.
Blinding	Blinding was not performed as subjective analysis was not needed and no group allocation was performed for structural experiments and electrophysiology.

Reporting for specific materials, systems and methods

We require information from authors about some types of materials, experimental systems and methods used in many studies. Here, indicate whether each material, system or method listed is relevant to your study. If you are not sure if a list item applies to your research, read the appropriate section before selecting a response.

Materials & experimental systems

n/a	Involved in the study
x	Antibodies
	x Eukaryotic cell lines
x	Palaeontology and archaeology
x	Animals and other organisms
x	Clinical data
x	Dual use research of concern

Methods

n/a	Involved in the study
x	ChIP-seq
x	Flow cytometry
x	MRI-based neuroimaging

Eukaryotic cell lines

Policy information about cell lines and Sex and Gender in Research

Cell line source(s)	SF9 cells from Invitrogen.<br>HEK 293T and HEK 293S GnTi- cells from American Type Culture Collection (ATCC).
Authentication	Cell lines were directly purchased from Invitrogen and ATCC. Cell line authentication was not performed at our hand during cell culture.
Mycoplasma contamination	Cell lines had been tested negative for mycoplasma contamination by Invitrogen and ATCC before purchase. They were not tested at our hand during cell culture.
Commonly misidentified lines<br>(See ICLAC register)	No commonly misidentified cell lines were used in the study.
